# Tooth Loss as a Predictor of Coronary Artery Disease Severity in Patients with Acute Myocardial Infarction: A Prospective Cross-Sectional Study

**DOI:** 10.3390/jcm15020610

**Published:** 2026-01-12

**Authors:** Corina Cinezan, Camelia Bianca Rus, Alexandra Cinezan, Gabriela Ciavoi

**Affiliations:** 1 Department of Medical Disciplines, Faculty of Medicine and Pharmacy, University of Oradea, 410073 Oradea, Romania; rus.cameliabianca@student.uoradea.ro; 2 Clinical County Emergency Hospital Bihor, 410169 Oradea, Romania; 3 Doctoral School of Biological and Biomedical Sciences, University of Oradea, 410087 Oradea, Romania; 4 Faculty of Dental Medicine, University of Medicine and Pharmacy, 400012 Cluj-Napoca, Romania; cinezan.alexandra@elearn.umfcluj.ro; 5 Department of Dental Medicine, Faculty of Medicine and Pharmacy, University of Oradea, 410068 Oradea, Romania; gciavoi@uoradea.ro

**Keywords:** tooth loss, periodontal disease, oral-systemic health, coronary artery disease, multivessel disease, acute myocardial infarction, cardiovascular risk, angiography, systemic inflammation

## Abstract

**Background:** Tooth loss reflects cumulative oral inflammation and has been associated with adverse cardiovascular outcomes. This study evaluated the relationship between the number of missing permanent teeth and the angiographic severity of coronary artery disease (CAD) in patients with acute myocardial infarction (AMI). **Methods:** In this prospective cross-sectional study, 200 consecutive AMI patients underwent coronary angiography and standardized dental assessment during hospitalization. Tooth loss was categorized as 1–10, 11–20, or 21–32 missing teeth. CAD severity was defined by the number of major epicardial arteries with significant stenosis. Multivariate logistic regression adjusted for age, sex, smoking status, diabetes, obesity, dyslipidemia, and hypertension. **Results:** Increasing tooth loss was associated with more extensive CAD. The mean number of affected vessels rose from 1.58 ± 0.79 in the 1–10 tooth-loss group to 2.06 ± 0.99 in the 21–32 group (*p* = 0.014). Tooth loss correlated with CAD severity (r = 0.19, *p* = 0.007). After adjustment, >20 missing teeth remained an independent predictor of multivessel disease (OR = 1.84; 95% CI: 1.01–3.34; *p* = 0.047). ROC analysis showed modest discrimination (AUC = 0.61). **Conclusions:** Extensive tooth loss independently correlates with greater angiographic CAD severity in AMI patients. Dental status may serve as a simple, non-invasive clinical marker of cardiovascular disease burden.

## 1. Introduction

Cardiovascular disease (CVD) remains the leading cause of death worldwide, accounting for nearly 17.9 million deaths annually, which represents about one-third of all global mortality [1]. At the same time, poor oral health, particularly periodontal disease and tooth loss, constitutes a major global health problem, affecting billions of individuals and ranking among the most prevalent chronic conditions [2]. Severe periodontitis alone affects up to 11% of the world’s adult population, and both periodontitis and tooth loss are strongly influenced by shared risk factors such as smoking, diabetes mellitus, obesity, hypertension, and socioeconomic disadvantage [3,4].

Tooth loss, the ultimate consequence of advanced periodontal disease, extends beyond being a marker of impaired oral function. It also reflects cumulative exposure to systemic inflammation and health inequalities, making it a visible surrogate for broader disease burden. Numerous epidemiological studies and meta-analyses have shown that individuals with extensive tooth loss face higher risks of myocardial infarction, stroke, and cardiovascular mortality [5,6,7]. A recent systematic review concluded that tooth loss is independently associated with cardiovascular disease mortality, reinforcing its role as a potential prognostic indicator [5].

The biological plausibility of this association is supported by several mechanisms. Periodontitis triggers systemic release of inflammatory mediators such as C-reactive protein, interleukin-6, and tumor necrosis factor-α, all of which are established contributors to endothelial dysfunction and atherosclerosis [8,9]. In addition, periodontal pathogens including Porphyromonas gingivalis have been identified in atherosclerotic plaques, supporting the hypothesis of direct bacterial dissemination into the vascular system [10,11]. Together, these findings underscore the oral-systemic link and suggest that poor oral health may actively contribute to atherogenesis and plaque instability.

Beyond atherogenesis, chronic systemic inflammation also contributes to adverse outcomes following coronary interventions. Inflammatory pathways have been implicated in neointimal hyperplasia and stent restenosis, limiting the long-term effectiveness of angioplasty and stent implantation and increasing the need for repeat revascularization procedures. Periodontal disease–related systemic inflammation may therefore not only influence the extent of coronary artery disease but also affect post-interventional outcomes through similar inflammatory mechanisms [12].

Despite growing recognition of this connection, few studies have directly examined the correlation between the extent of tooth loss and the angiographic severity of coronary artery disease, particularly in patients with acute myocardial infarction. Establishing such an association could have important clinical implications, as the number of missing teeth represents an easily observable, inexpensive, and non-invasive parameter. If validated, tooth loss could supplement traditional cardiovascular risk assessment and serve as a practical marker of severe coronary involvement.

The present study aims to investigate the association between the number of missing teeth and the angiographic severity of CAD in patients admitted with acute myocardial infarction. By quantifying coronary involvement through coronary angiography and correlating it with dental status, this research seeks to provide novel insights into the oral-systemic axis and to evaluate whether tooth loss may serve as a readily available clinical marker of cardiovascular disease burden.

Recognizing the mouth as a window to systemic health has important public health implications. Integrating oral assessment into cardiovascular care may facilitate early identification of individuals at elevated risk.

## 2. Materials and Methods

### 2.1. Study Design and Setting

This was a prospective, observational, cross-sectional study conducted at the Clinical County Emergency Hospital Bihor, Romania, an emergency care and tertiary referral center, between November 2024 and September 2025. The study protocol was reviewed and approved by the Institutional Ethics Committee of the Clinical County Emergency Hospital Bihor and all procedures adhered to the ethical principles outlined in the Declaration of Helsinki. Written informed consent was obtained from all participants prior to enrollment.

### 2.2. Study Population

A total of 200 consecutive patients were recruited from the Cardiology Department of Clinical County Emergency Hospital Bihor during hospitalization for acute myocardial infarction. Both ST-elevation myocardial infarction (STEMI) and non-ST-elevation myocardial infarction (NSTEMI) were eligible for inclusion. The diagnosis of AMI was established based on current European Society of Cardiology (ESC) guidelines, requiring typical clinical presentation, elevated cardiac biomarkers, and confirmatory electrocardiographic changes.

The sample size (n = 200) was determined based on feasibility and available consecutive admissions during the study period, while ensuring adequate statistical power to detect a weak-to-moderate correlation (r = 0.20) between the number of missing teeth and coronary artery disease severity. This expected effect size was chosen based on previously published studies reporting similar magnitudes of association between oral health parameters and angiographic coronary disease severity. With α = 0.05 and 1 − β = 0.80, the calculated sample size was sufficient to detect such an association. Post hoc power analysis confirmed adequate precision for the observed effect size.

Inclusion criteria were: age ≥ 18 years, confirmed diagnosis of AMI (STEMI or NSTEMI), undergoing coronary angiography during the index hospitalization.

Exclusion criteria were: history of valvular disease and of congenital heart disease, prior myocardial infarction, prior coronary artery bypass grafting, prior percutaneous transluminal coronary angioplasty, known chronic inflammatory or autoimmune disease (including rheumatoid arthritis, systemic lupus erythematosus, inflammatory bowel disease, ankylosing spondylitis, psoriasis, or other systemic inflammatory conditions requiring chronic anti-inflammatory or immunosuppressive therapy), acute infection, inability or refusal to provide informed consent, prior third molar extraction for orthodontic purposes.

### 2.3. Dental Assessment

Dental examinations were performed by two cardiologists and a senior dentistry student who received specific training from a senior dentist prior to study initiation. It was performed during hospitalization, within the first 72 h of admission. The number of permanently missing teeth was recorded for each patient using a standardized oral examination protocol under appropriate lighting. A full dentition was defined as 32 teeth. Where uncertainty existed, dental history was cross-checked with available medical and dental records. Patients were categorized into groups according to the number of missing teeth:

- 1–10 teeth lost
- 11–20 teeth lost
- 21–32 teeth lost

### 2.4. Angiographic Evaluation of CAD Severity

All patients underwent diagnostic coronary angiography using standard femoral or radial artery access with the Judkins technique. CAD severity was defined based on the number of major coronary arteries affected by significant stenosis. Significant stenosis was defined as ≥50% luminal narrowing for the left main coronary artery (LCA) and ≥70% for other major epicardial vessels: left anterior descending artery (LAD), circumflex artery (XCA) and right coronary artery (RCA), in accordance with established coronary angiography criteria and current clinical guidelines [13].

Patients were classified into the following categories:

- Single-vessel disease (1-VD): one major epicardial artery with significant stenosis
- Two-vessel disease (2-VD): two major epicardial arteries with significant stenosis
- Three-vessel disease (3-VD): three or more major epicardial arteries with significant stenosis

### 2.5. Data Collection and Covariates

Baseline demographic and clinical characteristics were obtained through structured interviews and medical record review. Data included age, sex, smoking status, presence of diabetes mellitus, hypertension, dyslipidemia, obesity, and other comorbidities. Laboratory values, echocardiographic findings, and angiographic data were also collected.

### 2.6. Statistical Analysis

All data were entered into a secure database and analyzed using SPSS version 20.0 (IBM Corp., Armonk, NY, USA) and Microsoft Excel 2010. Continuous variables were expressed as mean ± standard deviation (SD), while categorical variables were presented as absolute numbers and percentages.

Comparisons between groups were made using Student’s *t*-test for continuous variables and the chi-square (χ^2^) test for categorical variables. Analysis of variance (ANOVA) was used for comparing mean values across multiple groups. Correlations between the number of missing teeth (NLPT) and the number of diseased coronary arteries were assessed using Pearson’s correlation coefficient.

Receiver operating characteristic (ROC) curves were constructed to evaluate the diagnostic performance of tooth loss in predicting multivessel coronary artery disease (CAD) and the area under the ROC curve (AUC) was used to quantify discriminative capacity. A two-tailed *p*-value < 0.05 was considered statistically significant.

Data distribution was tested for normality using the Shapiro–Wilk test. Continuous variables with normal distribution are presented as mean ± standard deviation (SD), whereas non-normally distributed variables are reported as median and interquartile range (IQR). For variables with large SDs, both mean ± SD and median (IQR) are provided in the corresponding tables.

## 3. Results

### 3.1. Baseline Characteristics

The study included 200 patients with acute myocardial infarction (AMI), of whom 134 (67.0%) were male and 66 (33.0%) female.

The mean age was 65.6 ± 9.3 years (range 25–88). Most participants were older than 60 years (73.0%).

Cardiovascular risk factors were highly prevalent, with hypertension in 75.5%, obesity in 46.0%, dyslipidemia in 30.5%, diabetes mellitus in 29.0%, and active smoking in 41.5% of patients.

Nearly all subjects (97.0%) had at least one conventional risk factor for coronary artery disease.

Baseline characteristics of the patients enrolled and their risk factors for CAD are presented in Table 1.

Data on other chronic inflammatory diseases were not systematically collected; however, based on available clinical histories, such conditions appeared infrequent in this population (<10%) and unlikely to affect the main associations observed.

### 3.2. Dental Status

The mean number of missing permanent teeth was 22.1 ± 9.8. Patients were categorized as follows:

1–10 missing teeth (19.0%), 11–20 missing teeth (17.0%) and 21–32 missing teeth (64.0%).

The mean NPLT was significantly higher in women than in men (24.6 ± 8.9 vs. 20.6 ± 10.2, *p* = 0.011).

Tooth loss increased progressively with age, from 7.5 ± 7.3 teeth in patients under 50 years to 29.0 ± 3.9 in those aged ≥80 years (*p* < 0.001).

Table 2 illustrates these data.

Although detailed cause-specific data were not available for all patients, clinical and historical assessment suggested that periodontal disease was the predominant cause of tooth loss in most cases, while dental caries accounted for a smaller proportion.

### 3.3. Coronary Angiographic Findings

All patients underwent coronary angiography during hospitalization.

The most frequently affected coronary artery was the left anterior descending (LAD) artery (71.0%), followed by the right coronary artery (RCA, 53.0%) and the circumflex artery (XCA, 41.5%).

Single-vessel disease (1-VD) was present in 86 patients (43.0%), two-vessel disease (2-VD) in 59 (29.5%), and three-vessel or more extensive disease (≥3-VD) in 55 (27.5%).

These findings are illustrated in Table 3.

### 3.4. Association Between Tooth Loss and CAD Severity

The mean number of affected coronary arteries increased progressively with the number of missing teeth, from 1.58 ± 0.79 in patients with 1–10 missing teeth to 2.06 ± 0.99 in those with 21–32 missing teeth (*p* = 0.014). A direct correlation was found between NLPT and the number of diseased coronary arteries (Pearson’s r = 0.19, *p* = 0.007).

In unadjusted analysis, patients with more than 20 missing teeth were 2.2 times more likely to present multivessel CAD (≥3 affected arteries) compared with those with fewer than 21 missing teeth (OR = 2.22, 95% CI: 1.10–4.48, *p* = 0.027).

Table 4 presents these findings.

### 3.5. Adjusted Analysis

To account for potential confounding, a multivariate logistic regression model was constructed with multivessel CAD as the dependent variable and NLPT category (≤20 vs. >20 teeth lost) as the primary independent variable.

Age, sex, smoking, diabetes, obesity, dyslipidemia, and hypertension were included as covariates.

After adjustment, extensive tooth loss (>20 missing teeth) remained independently associated with multivessel CAD (adjusted OR = 1.84, 95% CI: 1.01–3.34, *p* = 0.047).

Among covariates, age > 65 years (*p* < 0.001) and diabetes mellitus (*p* = 0.015) were also significant predictors of multivessel disease. Table 5 illustrates these data.

**Figure 1:**
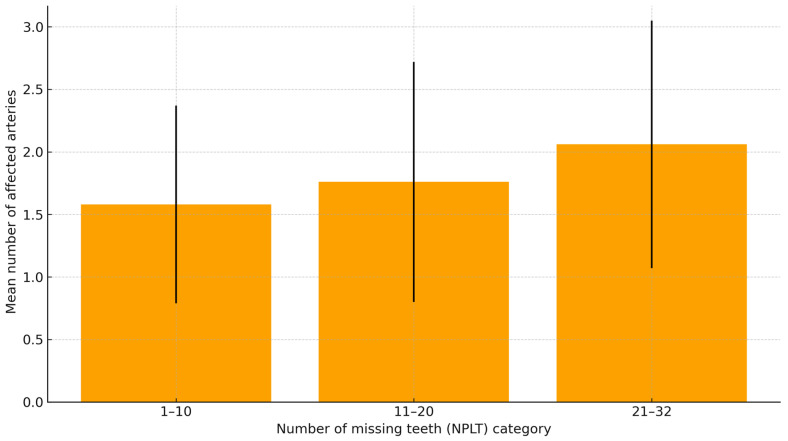
Severity of coronary artery disease (mean number of affected vessels ± SD) according to tooth-loss category.

As shown in Figure 1, CAD severity increased progressively with the number of missing teeth.

**Figure 2:**
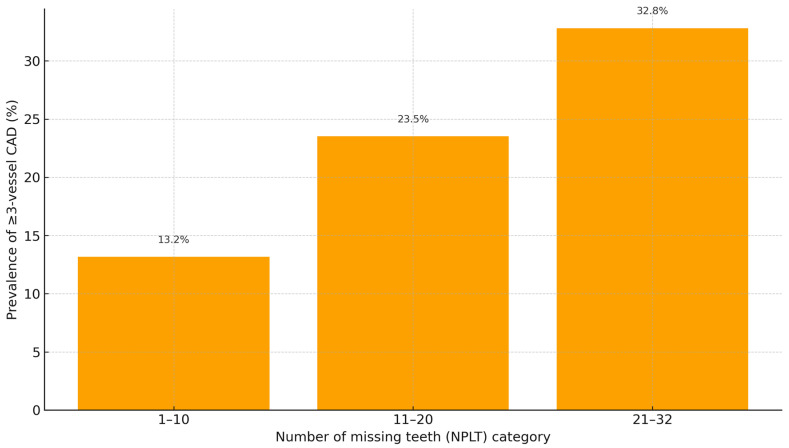
Prevalence (%) of multivessel (≥3-vessel) coronary artery disease by tooth-loss category.

Figure 2 demonstrates that multivessel disease was most prevalent among patients with >20 missing teeth.

**Figure 3:**
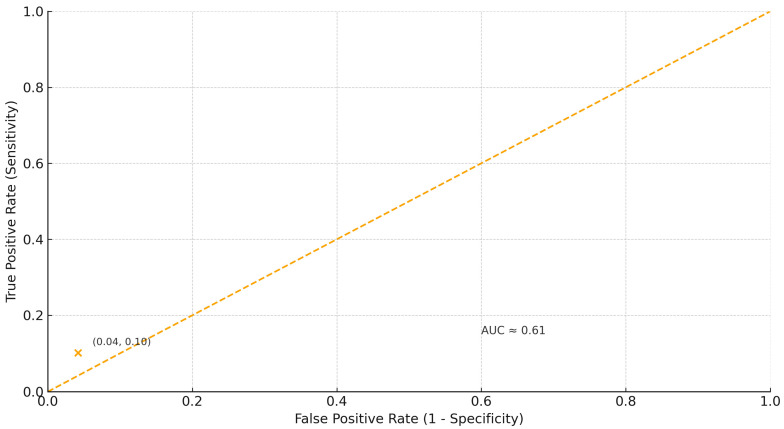
Receiver operating characteristic (ROC) curve of the number of lost teeth (NLPT) for predicting multivessel CAD (AUC = 0.61).

Figure 3 presents the ROC curve illustrating the moderate discriminative ability of NLPT for predicting severe CAD (AUC = 0.61).

### 3.6. Diagnostic Performance

ROC analysis evaluated the discriminative ability of NPLT for identifying multivessel CAD. The area under the ROC curve (AUC) was 0.61 (95% CI 0.52–0.70, *p* = 0.048), indicating modest predictive value. A threshold of >20 missing teeth demonstrated high specificity (95.8%) but low sensitivity (10.2%) for identifying severe coronary involvement. The positive predictive value was 81.3%, while the negative predictive value was 37.5%.

## 4. Discussion

This study demonstrates an independent association between the number of missing permanent teeth and the angiographic severity of coronary artery disease in patients presenting with acute myocardial infarction. Even after adjusting for age, sex, and major cardiovascular risk factors such as smoking, diabetes, obesity, dyslipidemia, and hypertension, patients with more than 20 missing teeth were significantly more likely to have multivessel CAD (adjusted OR = 1.84, 95% CI: 1.01–3.34, *p* = 0.047). Although the association was modest in strength, it persisted after controlling for key confounders, suggesting that tooth loss may act as an independent clinical marker of systemic vascular burden.

The periodontal status of dentate patients was not systematically quantified using standardized indices like probing depth, attachment loss or bleeding on probing. Nevertheless, clinical evaluation indicated that a substantial proportion exhibited signs of chronic periodontal inflammation. This residual inflammation could plausibly contribute to systemic inflammatory load and exacerbate cardiovascular disease, whereas in edentulous patients the bacterial burden associated with active periodontitis is absent. Therefore, the observed association between tooth loss and CAD severity may partly underestimate the contribution of ongoing periodontal inflammation among partially dentate patients.

### 4.1. Comparison with Previous Studies

The results of our investigation are consistent with prior epidemiological studies and meta-analyses that have linked tooth loss and periodontitis to an increased risk of myocardial infarction, stroke, and cardiovascular mortality [5,6,7]. For example, Elter et al. [14] found that individuals missing more than nine teeth had a higher risk of multivessel CAD. Ylostalo et al. [15] identified a relationship between the number of missing teeth and elevated C-reactive protein levels, suggesting a potential systemic inflammatory link. Furthermore, Holmlund et al. [16] reported that individuals with fewer than ten remaining teeth had significantly higher cardiovascular mortality compared with those retaining more than twenty-five teeth. Lee et al. [17] emphasized that the number of lost teeth was significantly associated with obstructive CAD, with patients who had obstructive CAD having significantly more lost teeth than patients without obstructive CAD. In addition, Donders et al. [18] showed that elevated coronary artery calcium scores were linked to tooth loss. Nonetheless Gao et al. [19] demonstrated an association between coronary heart disease, periodontitis and the number of teeth. Shen et al. [20] found that the number of missing teeth correlates with the severity of coronary atherosclerosis. Similarly, Aminoshariae et al. [5] demonstrated through a large meta-analysis that tooth loss was an independent predictor of cardiovascular disease mortality. Our findings add to this growing body of evidence by demonstrating that not only does tooth loss predict adverse outcomes, but it also correlates directly with the angiographic extent of CAD in the acute setting of AMI, a relationship less frequently reported in the literature [21,22].

### 4.2. Potential Mechanisms

Several biological mechanisms may explain the observed relationship between tooth loss and CAD severity. Periodontal disease, the leading cause of tooth loss, is characterized by chronic local infection and systemic inflammation. Elevated levels of inflammatory mediators such as C-reactive protein, interleukin-6, and tumor necrosis factor-α contribute to endothelial dysfunction, promote lipid deposition, and accelerate atherogenesis [8,9]. Moreover, oral pathogens including Porphyromonas gingivalis and Aggregatibacter actinomycetemcomitans have been detected within atherosclerotic plaques [10,11], suggesting that bacteremia originating from the oral cavity may directly contribute to vascular inflammation and plaque instability. These microorganisms may promote macrophage activation, oxidative stress, and foam cell formation, thereby accelerating atherosclerotic burden and contributing to multivessel coronary disease. Additionally, periodontal inflammation may alter lipid metabolism, enhance oxidative stress, and trigger immune dysregulation, further promoting vascular injury [23,24].

Tooth loss may also reveal unfavorable health behaviors and socioeconomic disadvantage, which themselves contribute to poor cardiovascular outcomes. Individuals with extensive tooth loss often exhibit poorer nutritional intake, increased consumption of soft, carbohydrate-rich foods, and reduced intake of fiber, fruits, and antioxidants, all of which may further exacerbate dyslipidemia and insulin resistance. Moreover, tooth loss is strongly associated with smoking, diabetes mellitus, and lower socioeconomic status, factors that independently and synergistically contribute to more diffuse and severe coronary artery involvement [19,25]. Smoking, poor nutrition, and limited access to medical and dental care are strongly associated with both oral disease progression and CAD. Thus, the relationship between tooth loss and CAD is likely multifactorial, involving direct biological effects of oral infection and inflammation as well as indirect influences through shared behavioral and social determinants of health [23].

From a temporal perspective, tooth loss represents a cumulative marker of chronic inflammatory burden over decades, whereas acute myocardial infarction reflects the final clinical manifestation of progressive atherosclerosis. Thus, extensive tooth loss may serve as a surrogate indicator of prolonged systemic inflammatory exposure, explaining its association with more extensive angiographic coronary artery disease in the acute setting [26,27].

Taken together, these mechanisms suggest that the relationship between tooth loss and CAD severity is multifactorial, involving direct inflammatory and microbial pathways as well as indirect effects mediated by shared risk factors and social determinants of health.

On the other hand, the present analysis also highlights the limitations of using tooth loss as a diagnostic tool. The ROC analysis yielded an AUC of 0.61, indicating only moderate discriminative capacity, with high specificity (95.8%) but low sensitivity (10.2%). This means that while the presence of extensive tooth loss strongly suggests advanced coronary disease, many patients with severe CAD will not be identified by this parameter alone. Therefore, tooth loss should not be viewed as a screening test but rather as a readily observable, low-cost marker that may supplement cardiovascular risk assessment in clinical practice.

### 4.3. Clinical Implications

Dental status is easily assessable, non-invasive, and reflects cumulative exposure to systemic inflammation, lifestyle and socioeconomic factors. Identifying individuals with significant tooth loss during routine clinical encounters may prompt more comprehensive cardiovascular evaluation, especially in primary care or resource-limited settings. Moreover, evidence suggests that treatment of periodontal disease can reduce systemic inflammation and improve endothelial function [16,24], underscoring the bidirectional benefit of oral and cardiovascular health management.

In our opinion, this research carries particular importance for patients with acute myocardial infarction. The simple observation of dental status, specifically, the extent of tooth loss, may provide valuable, immediate insight into the probable severity of underlying coronary artery disease. Patients with significant tooth loss are more likely to present with multivessel involvement and, consequently, to experience a more complex clinical course with higher risk of complications. Recognizing this association allows clinicians to identify vulnerable individuals at the bedside through a non-invasive and easily assessable marker.

From a practical standpoint, such patients warrant heightened clinical vigilance, early multidisciplinary evaluation, and potentially greater allocation of both human and technical resources to optimize their management. Integrating this observation into cardiovascular care encourages a more holistic view of disease burden, highlighting the mouth as a mirror of systemic vascular health. This perspective strengthens the argument for interdisciplinary collaboration between cardiology and dentistry and supports the inclusion of oral health evaluation as part of comprehensive cardiac risk stratification and post-infarction follow-up.

From a preventive healthcare perspective, simple clinical markers such as the number of missing teeth may serve as accessible indicators of cumulative cardiovascular risk, particularly in settings with limited resources or reduced access to advanced diagnostics.

While previous studies have documented associations between poor oral health and cardiovascular outcomes, our study adds specific clinical granularity by focusing on patients with acute myocardial infarction and by linking dental status to angiographically verified CAD severity. Within the context of acute myocardial infarction, the consistent relationship between tooth loss and CAD severity remains clinically relevant and warrants further exploration in prospective, multicenter studies.

This approach provides a direct and objective measure of coronary disease burden, complementing the predominantly epidemiological literature. Furthermore, the inclusion of a Central-Eastern European population offers region-specific data from an area underrepresented in oral–systemic research.

Although tooth loss is a simplified oral parameter, it serves as a cumulative indicator of chronic inflammatory exposure and impaired oral function, which together mirror long-term systemic health trajectories.

### 4.4. Strengths of the Study

The most important strength of our study is integration of dental and cardiovascular assessment; this study uniquely combines comprehensive dental evaluation (number of missing permanent teeth) with coronary angiography, the gold standard for assessing CAD severity, providing direct clinical evidence for the oral–systemic link.

Another important strength is adjustment for major confounders: multivariate logistic regression models accounted for age, sex, smoking, diabetes mellitus, obesity, dyslipidemia, and hypertension, minimizing bias and supporting the independence of the observed association.

Our study has well-characterized clinical population. It included a consecutive series of 200 acute myocardial infarction patients, ensuring real-world representativeness of the sample and reducing selection bias.

Robust statistical analysis is another point of interest, both correlation and regression analyses were performed. The diagnostic performance of tooth loss was evaluated using ROC curve analysis, demonstrating methodological transparency and reproducibility.

The research has clinical applicability: tooth loss is an easily observable, non-invasive, and low-cost indicator that may help clinicians identify patients at higher cardiovascular risk during routine medical or dental visits.

Interdisciplinary relevance is another important feature of our findings. The research bridges cardiology and dentistry, supporting a multidisciplinary approach to cardiovascular prevention and reinforcing the systemic implications of oral health.

### 4.5. Limitations of the Study

There are some limitations of our research.

Cross-sectional design is one of them. The observational nature of the study precludes establishing a causal relationship between tooth loss and CAD severity. It was a single-center study, conducted in a single tertiary care institution, which may limit generalizability to broader or more diverse populations. Nevertheless, within the context of acute myocardial infarction, the consistent relationship between tooth loss and CAD severity remains clinically relevant and warrants further exploration in prospective, multicenter studies.

Another limitation of our study is the lack of detailed periodontal assessment: data on periodontal status, plaque index, and microbiological markers were not collected, preventing a deeper mechanistic analysis. There are potential residual confounding: despite adjustment for major cardiovascular risk factors, unmeasured factors such as socioeconomic status, dietary habits, oral hygiene practices, or genetic predisposition could influence results.

The research has limited diagnostic power; the ROC analysis showed moderate discriminative ability (AUC = 0.61), indicating that tooth loss should be considered an indicator rather than a diagnostic test. Sample size is modest. Although adequate for detecting moderate associations, larger multicenter studies are required to validate these findings and improve statistical precision.

### 4.6. Future Directions

Future multicenter studies with larger populations, detailed periodontal evaluations, and longitudinal follow-up are needed to confirm these findings. Incorporating markers of systemic inflammation, microbiome profiling, and standardized periodontal indices would strengthen causal inference. Interventional trials examining whether periodontal therapy can reduce cardiovascular events would also provide valuable clinical insights. Moreover, integrating oral health variables into established cardiovascular risk prediction models could be a promising direction for precision prevention strategies.

## 5. Conclusions

In this prospective cross-sectional study of patients with acute myocardial infarction, extensive tooth loss was independently associated with greater angiographic severity of coronary artery disease. Patients with more than 20 missing teeth were significantly more likely to present with multivessel coronary involvement, even after adjustment for traditional cardiovascular risk factors. Although the discriminative capacity of tooth loss was modest, its high specificity suggests that extensive tooth loss may serve as a simple, readily observable marker of advanced coronary disease burden in the acute clinical setting.

This study reinforces the emerging evidence that oral health is intricately linked to cardiovascular integrity. It reinforces the need for interdisciplinary collaboration between cardiology and dentistry, promoting a holistic healthcare model that addresses shared risk factors and systemic inflammation pathways. By demonstrating an independent association between tooth loss and angiographic coronary artery disease severity in patients with acute myocardial infarction, our work underscores the diagnostic and prognostic potential of dental indicators within systemic disease frameworks.

We hope this research will stimulate further interdisciplinary collaboration and inspire new diagnostic strategies aimed at uniting oral and cardiovascular health for improved patient outcomes.

## Figures and Tables

**Table 1:** Baseline characteristics of the study population (n = 200).

Variable	Total n (%) or Mean ± SD
Age (years)	65.6 ± 9.3
<60 years	54 (27.0%)
≥60 years	146 (73.0%)
Male	134 (67.0%)
Female	66 (33.0%)
Smoking	83 (41.5%)
Diabetes mellitus	58 (29.0%)
Obesity	92 (46.0%)
Dyslipidemia	61 (30.5%)
Hypertension	151 (75.5%)
≥1 risk factor present	194 (97.0%)

Note: Values are presented as mean ± standard deviation (SD) for normally distributed variables and as number (percentage) for categorical variables. No between-group comparisons were conducted for this descriptive table.

**Table 2:** Dental characteristics according to demographic variables.

Variable	1–10 Lost Teeth	11–20 Lost Teeth	21–32 Lost Teeth	*p* Value
Sex				
Male (n = 134)	30 (22.4%)	24 (17.9%)	80 (59.7%)	0.011
Female (n = 66)	8 (12.1%)	10 (15.2%)	48 (72.7%)	
Age group				<0.001
<50 years	16 (80.0%)	3 (15.0%)	1 (5.0%)	
50–59 years	12 (35.3%)	10 (29.4%)	12 (35.3%)	
60–69 years	6 (9.8%)	11 (18.0%)	44 (72.1%)	
70–79 years	4 (5.9%)	10 (14.7%)	54 (79.4%)	
≥80 years	0 (0.0%)	0 (0.0%)	17 (100.0%)	

Note: Values are expressed as number (percentage). *p*-values were calculated using the chi-square (χ^2^) test to compare distributions across tooth-loss categories.

**Table 3:** Angiographic characteristics of CAD.

Parameter	n (%)
1-vessel disease	86 (43.0%)
2-vessel disease	59 (29.5%)
≥3-vessel disease	55 (27.5%)
LAD (left anterior descending)	142 (71.0%)
RCA (right coronary artery)	106 (53.0%)
XCA (circumflex artery)	83 (41.5%)
LCA (left main)	20 (10.0%)

Note: Values are shown as number (percentage). This table is descriptive; therefore, no statistical comparisons were performed.

**Table 4:** Association between number of missing teeth and CAD severity.

NLPT Group	1-Vessel	2-Vessel	≥3-Vessel	Mean No. of Affected Arteries ± SD	*p* Value
1–10 teeth lost (n = 38)	22 (57.9%)	11 (28.9%)	5 (13.2%)	1.58 ± 0.79	
11–20 teeth lost (n = 34)	18 (52.9%)	8 (23.5%)	8 (23.5%)	1.76 ± 0.96	
21–32 teeth lost (n = 128)	46 (35.9%)	40 (31.3%)	42 (32.8%)	2.06 ± 0.99	0.014

Note: Values are expressed as number (percentage) unless otherwise indicated. Mean number of affected coronary arteries is presented as mean ± SD. The *p*-value refers to a one-way analysis of variance (ANOVA) comparing the three tooth-loss categories.

**Table 5:** Multivariate logistic regression for predictors of multivessel CAD (≥3 affected arteries).

Variable	Adjusted OR	95% CI	*p* Value
>20 missing teeth	1.84	1.01–3.34	0.047
Age > 65 years	2.62	1.44–4.78	0.001
Male sex	1.28	0.72–2.27	0.395
Smoking	1.34	0.73–2.46	0.341
Diabetes mellitus	2.05	1.15–3.65	0.015
Obesity	1.16	0.66–2.05	0.604
Dyslipidemia	1.09	0.59–2.02	0.774
Hypertension	1.12	0.59–2.11	0.739

Note: *p*-values, adjusted odds ratios (OR), and 95% confidence intervals (CI) were derived from multivariate logistic regression including age, sex, smoking status, diabetes mellitus, obesity, dyslipidemia, and hypertension as covariates.

## Data Availability

The raw data supporting the conclusions of this article will be made available by the authors on request. The data are not publicly available due to privacy reasons.
